# NLRP3 Inflammasome in Metabolic-Associated Kidney Diseases: An Update

**DOI:** 10.3389/fimmu.2021.714340

**Published:** 2021-07-08

**Authors:** Wei Xiong, Xian-Fang Meng, Chun Zhang

**Affiliations:** ^1^ Department of Nephrology, Union Hospital, Tongji Medical College, Huazhong University of Science and Technology, Wuhan, China; ^2^ Department of Neurobiology, School of Basic Medical Sciences, Tongji Medical College, Huazhong University of Science and Technology, Wuhan, China

**Keywords:** NLRP3, inflammasome, kidney diseases, metabolic syndrome, innate immunity

## Abstract

Metabolic syndrome (MS) is a group of complex metabolic disorders syndrome, which refers to the pathological state of metabolism disorder of protein, fat, carbohydrate and other substances in human body. The kidney is an important organ of metabolism, and various metabolic disorders can lead to the abnormalities in the structure and function of the kidney. The recognition of pathogenesis and treatment measures of renal damage in MS is a very important part for the renal function preserve. Inflammatory response caused by various metabolic factors is a protective mechanism of the body, but persistent inflammation will become a harmful factor and aggravate kidney damage. Inflammasomes are sensors of the innate immune system that play crucial roles in initiating inflammation in response to acute infections and chronic diseases. They are multiprotein complex composed of cytoplasmic sensors (mainly NLR family members), apoptosis-associated speck-like protein (ASC or PYCARD) and pro-caspase-1. After receiving exogenous and endogenous stimuli, the sensors begin to assemble inflammasome and then promote the release of inflammatory cytokines IL-1β and IL-18, resulting in a special way of cell death named pyroptosis. In the kidney, NLRP3 inflammasome can be activated by a variety of pathways, which eventually leads to inflammatory infiltration, renal intrinsic cell damage and renal function decline. This paper reviews the function and specific regulatory mechanism of inflammasome in kidney damage caused by various metabolic disorders, which will provide a new therapeutic perspective and targets for kidney diseases.

## Introduction

Metabolic syndrome (MS) is a group of complex metabolic disorders syndrome, which refers to the pathological state of metabolism disorder of protein, fat, carbohydrate and other substances in human body (1). In the 1990s, the overall prevalence of adult MS in the United States was 22%, and the prevalence increased with age, among which the prevalence rates of 20-29, 60-69 and over 70 years old were 6.7%, 43.5% and 42%, respectively (2). By the 2000’s, the prevalence continued to increase to 34.5% (3). The etiology of MS has not been clear, and it is considered to be the result of multi gene and multi environment interaction, which is closely related to genetics and immunity (4). The disease is affected by many environmental factors, mainly manifested in the high fat, high carbohydrate diet structure, low labor intensity and less exercise (4). MS includes a variety of metabolic disorders, including obesity, hyperglycemia, hypertension, dyslipidemia, high blood viscosity, high uric acid, high fatty liver incidence and hyperinsulinemia (5). At present, it is believed that the common causes of these factors are insulin resistance and hyperinsulinemia caused by obesity, especially central obesity (6). MS is a risk factor for a variety of diseases, such as hypertension, coronary heart disease, stroke, chronic kidney disease (CKD), and even some cancers, including breast cancer, endometrial cancer, prostate cancer related to sex hormone, as well as pancreatic cancer, hepatobiliary cancer, colon cancer in digestive system (6–8).

The kidney is an important organ of metabolism, and various metabolic abnormalities can affect the structure and function of kidney. Among people with MS, the prevalence of CKD exceeds 20%, which is much higher than that of the general population. At the same time, among patients with CKD, the prevalence of MS and subgroup metabolic disorders is much higher than that of non-CKD patients (9). A retrospective analysis of more than 6,000 American adults aged over 20 years found that MS was an independent risk factor for CKD (10). The probability of CKD and microalbuminuria was 2.6 times and 1. 9 times of that of the normal population, and the more abnormal components of MS metabolism, the greater the risk (10). A study of 75,468 Chinese people also supported the above view. The incidence of CKD in MS patients and those without MS was 57% and 28%, respectively (11). The clinical manifestations of MS related renal damage include glomerular hyperfiltration, microalbuminuria, proteinuria, changes in renal tubular function, eGFR < 60ml/(min·1.73m^2^), and increase of renal vascular resistance by ultrasonic. Insulin resistance is the central link of MS. Insulin receptors are widely expressed in the kidney, such as podocytes, mesangial cells, endothelial cells and renal tubular epithelial cells (12). Observation of kidney tissue pathology of donor kidneys revealed that chronic pathological changes were more common in kidney tissues of MS patients, manifested as varying degrees of glomerular sclerosis, renal tubular atrophy, renal interstitial fibrosis and arteries hardening (13).

Therefore, exploring the pathogenesis and prevention measures of renal damage in MS is a very important part of the prevention and treatment of CKD. This article summarizes the important role of inflammasome in renal damage caused by different metabolic factors, and provides a new perspective for the treatment of CKD in the future.

## General Introduction of NLRP3 Inflammasome

Nod like receptor protein 3 (NLRP3) inflammasome is a macromolecular polyprotein complex with a molecular weight of about 700 kDa, which has the function of regulating chronic inflammatory response. NLRP3 inflammasome consists of nucleotide-binding domain–like receptors (NLRs), apoptosis-associated speck-like protein containing caspase recruitment domain (ASC) and caspase protease. The structure of NLRs mainly includes the middle nucleotide-binding and oligomerization domain (NACHT), the downstream adapter protein pyrindomain (PYD) or caspase recruitment domain (CARD), and leucine-richrepeats (LRRs). Caspase-1 is the activated form of pro-caspase-1, which can cleave cytokine precursors such as interleukin (IL)-1β, IL-18 and other cytokine precursors, transform them into mature form, and participate in the inflammatory reaction (14).

The inflammasome is a complex composed of a variety of proteins in the cytoplasm, which integrates different damage stimulating signals and activates the innate immune defense function (14, 15). The innate immune system recognizes invading microorganisms and danger signals in the body through specific pattern recognition receptors (PRRs). Currently known PRRs are divided into two types, namely toll-like receptors (TLRs) located on the cell membrane and NLRs located in the cytoplasm (16). NLRP3 is the most well-studied and most comprehensive inflammasome in the family of NLRs. After LRRs of NLRP3 recognizes specific signal, it exposes and polymerizes the NACHT domain, and recruits ASC and pro-caspase-1 through PYD-PYD and CARD-CARD. Through the cleavage of pro-caspase-1 to mature caspase-1, cytokine precursors of IL-1β and IL-18 are cleaved into an active form and secreted out of the cell. In addition to promoting the maturation and secretion of IL-1β and IL-18, it can also mediate a special programmed cell death pyroptosis by activating caspase-1, which is characterized by the formation of caspase-1-dependent plasma membrane pore size, a large number of release of inflammatory mediators and DNA damage, and finally leads to osmotic disintegration of cells (17).

Many endogenous and exogenous factors can stimulate the production of NLRP3 inflammasome through different mechanisms. There are clear reports about crystals or particles (cholesterol crystals, asbestos, silica, etc.), bacterial toxins, microorganisms (viruses, bacteria and fungi), and some vaccine adjuvants (18, 19). Given that NLRP3 inflammasome is an intracellular recognition receptor and the diversity of recognition substances, these activators may have a common endogenous signal transduction molecule, however this common endogenous signal transduction molecule is not clear yet (20). At present, there are mainly three different modes to illustrate the activation mechanism of NLRP3 inflammasome. These three modes include potassium channel open and outflow, cathepsin-B secretion caused by lysosomal damage and rupture, and the production of reactive oxygen species (ROS) (21). Various microbial toxins, enzymes and extracellular ATP can activate ATP-P2X7 receptors, make potassium ions outflow, and activate NLRP3 inflammasome (22). Crystalline substances such as silicon dioxide, antibiotics and antifungal drugs activate inflammasome through ROS and cathepsin-B (23, 24).

**Figure 1:**
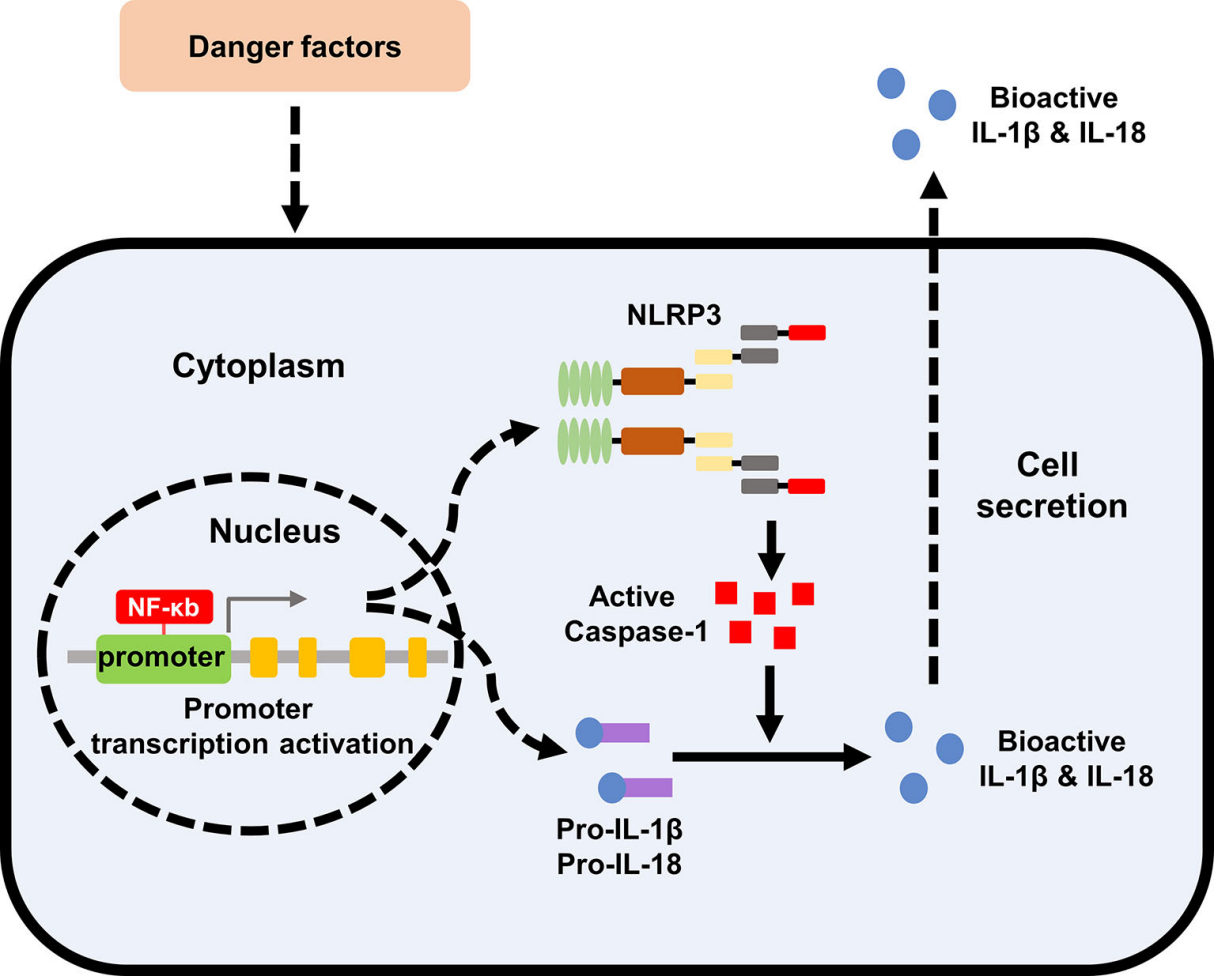
Formation and activation of NLRP3 inflammasome. The effect of extracellular stimulating factors activates the intracellular NF-κB pathway. The activation of NF-κB pathway promotes the expression of inflammasome NLRP3, IL-1β, and IL-18. The activation of the inflammasome NLRP3 promotes the activation of caspase-1, and the activated caspase-1 promotes the maturation of IL-1β and IL-18, which are then secreted into the extracellular to exert biological effects. NLRP3, nod like receptor protein 3; IL, interleukin.

Renal inflammatory response is the immune response of the kidney to infectious or non-infective activators. The specific expression of NLRP3 inflammasome components in kidney tissues has not yet been fully clarified. Renal mononuclear phagocytes, such as dendrites and macrophages, can express the components of NLRP3 inflammasome and may induce cell death by activating caspase-1 (25). At the same time, some studies have confirmed that renal tubular epithelial cells and podocytes also activate the NLRP3-ASC-caspase-1 axis, express and release mature IL-1β and IL-18 (26–28). As an intracellular pattern recognition receptor, NLRP3 inflammasome plays an important role in stimulating and regulating immune inflammation. The activation of NLRP3 inflammasome is involved in the acute and chronic inflammation of the kidneys by inducing the secretion of IL-1β and IL-18, leading to the automatic defense and inflammatory response (26, 29). NLRP3 inflammasome also participates in the occurrence and development of a variety of metabolic diseases as an important member (30). Therefore, the in-depth study on the mechanism of NLRP3 inflammasome associated with metabolic disorders and kidney injury will provide new ideas and directions for the treatment of metabolic related kidney diseases. The specific formation and activation of NLRP3 Inflammasome was shown in **Figure 1**.

## NLRP3 Inflammasome in Metabolic-Associated Kidney Diseases

### Diabetic Nephropathy

Diabetes mellitus (DM) is a group of metabolic diseases characterized by hyperglycemia. When DM continues to progress, it often causes chronic damage to the eyes, kidneys, blood vessels, and feet. Among them, diabetic nephropathy (DN) is the most harmful inflammatory complication. It is also the main microvascular of DM and the main cause of end-stage renal disease (ESRD). Inflammatory response is the key factor for the sustainable development of DN. The activation of various inflammatory factors, such as C-reactive protein (CRP), monocyte chemoattractant protein-1 (MCP-1) and inflammasomes, promote macrophage infiltration, renal tubular fibrosis, and eventually accelerate glomerulosclerosis (31).

The activation of NLRP3 inflammasome was detected in DN patients and diabetic mice (32). MCC950 was a selective and potent inhibitor of NLRP3 inflammasome, the use of which improved renal function, podocyte injury and renal fibrosis in db/db mice (33). Homozygous and hemizygous caspase-1 deficiency had protective effect on db/db mice, while caspase-3 deficiency had not, suggesting that caspase-3-dependent cell death had no significant effect on the formation of DN, while caspase-1-dependent inflammatory activation played an important role (34). Moreover, the use of a novel monoclonal antibody of IL-1β in diabetic mice reduced renal damage markers, ameliorated fibrosis, and preserved the number of podocytes (35). Thioredoxin-interacting protein (TXNIP) was a mediator of oxidative stress and has been reported to interact with NLRP3 inflammasome, leading to its activation (36). The expression of TXNIP and NLRP3 was both significantly increased in diabetic rats (36). Polyphenols, natural antioxidants, have been proved to reduce pyroptosis in DN, probably due to the inhibition of TXNIP/NLRP3 pathway (37). Other drugs with antioxidant function have also been confirmed to improve DN by targeting NLRP3 inflammasome, suggesting that NLRP3 inflammasome plays a crucial role in the pathogenesis of DN (38–40).

Podocytes, namely glomerular visceral epithelial cells, participate in stabilizing glomerular capillaries, maintaining the function of glomerular filtration barrier, regulating ultrafiltration coefficient K/f and maintaining the normal morphology of glomerular basement membrane (GBM) (41). Studies have shown that podocyte injury plays a key role in the pathogenesis of DN (41). High glucose (HG) activated the NLRP3 inflammasome in mouse podocytes, which was manifested by increased protein levels of NLRP3, ASC and caspase-1, and the activity of caspase-1 was also significantly elevated (42). After the podocytes were transfected with NLRP3-small interfering RNA (siRNA), the expression of caspase-1 and IL-1β was reduced, while the expression of the podocyte functional protein nephrin was significantly increased (43). The mechanism of NLRP3 inflammasome on podocytes has not been fully elucidated. It has been found that activation of NLRP3 inflammasome aggravated podocyte autophagy and reduced nephrin expression, while NLRP3 silencing effectively restored podocyte autophagy and alleviated podocyte injury induced by HG, suggesting that autophagy might participate in the regulation of NLRP3 inflammasome on podocytes (44).

Glomerular mesangial cells play an important role in the process of glomerular injury and repair. Early DN mainly manifested in the proliferation of mesangial cells, which then synthesize and secrete of a large number of mesangial matrixes, gradually occlude the capillaries and lead to glomerulosclerosis. It was found that the activation of NLRP3 also existed in glomerular mesangial cells stimulated by HG (45). Some extracts of traditional Chinese medicine have been found to target NLRP3 to alleviate HG induced mesangial cell proliferation (46, 47).

Renal tubular injury is one of the important determinants of progressive renal failure in DN. *In vitro*, the expression of NLRP3 and the release of IL-1β, IL-18 and ATP were significantly increased in HK-2 cells stimulated by HG (48). Knockdown of NLRP3 resisted HG induced tubular EMT by inhibiting ROS production and the phosphorylation of Smad3, p38MAPK and ERK1/2 (49). The overproduction of mitochondrial ROS (mtROS) plays a key role in inflammation. Treating HK-2 cells with the mtROS antioxidant MitoQ inhibited the dissociation of thioredoxin (TRX) from TXNIP, and then blocked the interaction between TXNIP and NLRP3, resulting in the inactivation of NLRP3 inflammasome and the inhibition of IL-1β maturation (50). Another study confirmed that ATP-P2X4 signaling mediated the activation of HG-induced NLRP3 inflammasome, regulated the secretion of IL-1β, and caused the development of tubulointerstitial inflammation in DN (48). IRE1α was endoplasmic reticulum stress (ERS)-related factor. Using IRE1α RNase specific inhibitor (STF-083010) in HG-induced NRK-52E cells inhibited the TXINP/NLRP3 pathway-mediated pyroptosis and renal damage, suggesting that ERS might also leading to the activation of NLRP3 inflammasome (51).

**Figure 2:**
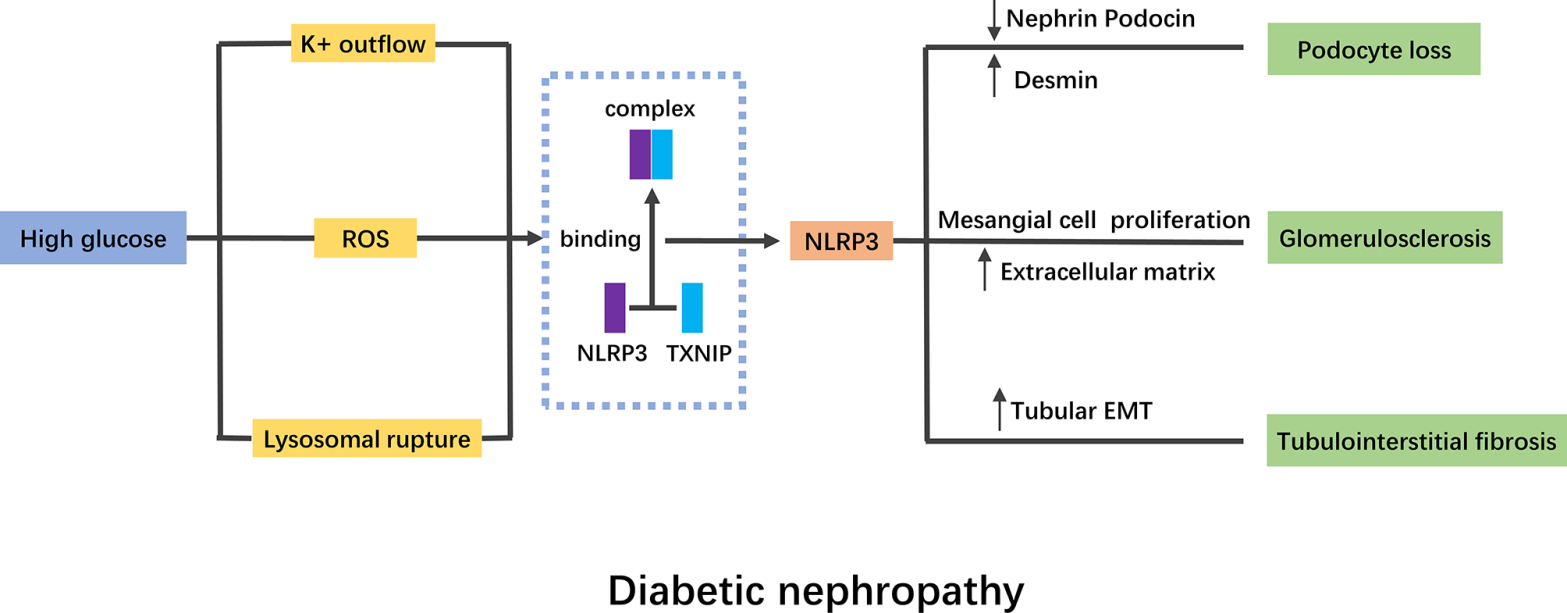
Mechanism of NLRP3 inflammasome in diabetic nephropathy. High glucose stimulation activates NLRP3 inflammasome mainly through K+ outflow, ROS and lysosomal rupture. TNXIP binding to NLRP3 is a pivotal mechanism of NLRP3 inflammasome activation. The activation of NLRP3 inflammasome will lead to podocyte lose, glomerulosclerosis and tubulointerstitial fibrosis. NLRP3, nod like receptor protein 3; ROS, reactive oxygen species; EMT, epithelial mesenchymal transition.

In general, HG stimulation can activate NLRP3 inflammasome through a variety of pathways, which will lead to the abnormalities of intrinsic cells in kidney (**Figure 2**). Current studies have proved that NLRP3 activation was widespread in DN, and targeted therapy of NLRP3 played an important role in the improvement of DN.

### Hypertension-Related Nephropathy

Hypertension-related nephropathy is the damage of renal structure and function caused by primary hypertension. The kidney can excrete excess water and sodium salt through urine, and prevent protein and blood cells from leaking out of blood vessels. High blood pressure increases the blood pressure in the blood vessels, leading to the leakage of protein into the urine, causing damage to the renal filter system. Long term poor control of hypertension will cause irreversible damage to the kidney. Clinical hypertension is related to kidney inflammation and increased circulating levels of IL-1β and IL-18, indicating that inflammasome activity may be involve in the blood pressure fluctuation and kidney injury (52).

In mice with deoxycorticosterone acetate and saline (1K/DOCA/salt)-induced hypertension, the mRNA levels of NLRP3, ASC, pro-caspase-1 and pro-IL-1β were evaluated, as well as the protein expression of active caspase-1 and mature IL-1β (53). ASC−/− mice exhibited a sluggish pressor response and the treatment of NLRP3 inflammasome inhibitor MCC950 reversed the hypertension in 1K/DOCA/salt treated mice (53). Nitric oxide (NO) inhibition and salt overload lead to hypertension, albuminuria, glomerulosclerosis, glomerular ischemia and interstitial fibrosis. In this model, allopurinol (ALLO), an NLRP3 inhibitor, significantly improved hypertension, proteinuria and interstitial inflammation and fibrosis (54). In a CKD model of 5/6 nephrectomy (5/6 Nx), the degree of tubulointerstitial fibrosis and proteinuria was decreased in NLRP3−/− mice, meanwhile, the mitochondrial morphology and CKD-related hypertension were also ameliorated (55). So far, some literatures have confirmed the important role of NLRP3 activation in hypertension-related nephropathy, but the specific regulatory mechanism still needs to be further explored.

### Obesity-Related Nephropathy

In 1974, Weisinger et al. firstly reported that severe obesity can lead to a large amount of proteinuria, and named this disease as obesity-related nephropathy (ORG) (56). Since then, clinical studies and animal experimental models have confirmed that obesity has a significant effect on the structure and function of the kidney (57). In recent years, although there are many studies on ORG, its specific pathogenesis is not fully understood. It is generally believed to be related to glucagon and insulin resistance, the role of adipocytokines, inappropriate activation of renin-angiotensin-aldosterone system (RAAS), release of inflammatory factors, lipid metabolism disorder and structural changes of kidney caused by obesity itself (58).

It was found that the mRNA levels and protein expressions of NLRP3, ASC and caspase-1 in renal cortex of ORG mice were significantly up-regulated (59). Also accompanied a significant increase by P2X7R, an activation molecule of NLRP3. The treatment of P2X7R antagonist (KN−62 or A438079) reversed the changes of NLRP3 inflammasome components as well as attenuated podocytes injury treated by leptin (59). The expression of IL-1β and IL-18 levels also gradually increased in the kidney of high-fat diet (HFD) fed mice detected by immunohistochemistry (60). Knockdown of caspase-1 expression with siRNA inhibited palmitate-induced death and apoptosis of HK-2 cells (60). Some natural substances and traditional Chinese medicine components have been shown to affect ORG by regulating the activation of NLRP3 inflammasome. Fisetin (FIS) is a natural flavonoid, which significantly attenuated HFD-induced histological changes in renal tissue samples, reduced the expression of kidney injury molecule-1 (KIM-1) and altered the expression of nephrin and podocin, thus improving renal insufficiency (61). In this process, the expression of NLRP3 inflammasome components was also decreased, suggesting that its mechanism might be related to inflammasome (61). Coptidis Rhizoma, a classical traditional Chinese herb, reduced dyslipidemia and improved urinary albumin to creatinine ratio and creatinine clearance rate in obesity-prone (OP) rats with high protein and high fat diet (62). The expression of NLRP3 inflammasome was also downregulated under Coptidis Rhizoma treatment (62).

At present, the research on the pathogenesis and treatment of ORG is relatively lacking, and the activation of NLRP3 may be an important link. Therefore, more extensive and in-depth research will give us a deeper understanding of ORG.

### Hyperuricemia

Uric acid is a kind of anionic organic acid which is slightly soluble in water. It is the end product of purine metabolism by xanthine oxidase. About 70% of uric acid in normal human body is excreted through kidney, and the remaining 30% is excreted through bile duct and intestine (63). The generation and excretion of uric acid in healthy human body is in dynamic balance. Once this balance is broken, the generation or excretion of uric acid increase or decrease, resulting in the accumulation of uric acid in the body, which will lead to hyperuricemia. Fasting serum uric acid level > 420 mmol · L ^- 1^ (male) and > 360 mmol · L ^- 1^ (female) is usually used as the diagnostic criteria of hyperuricemia.

Hyperuricemia can easily lead to renal hemodynamics, histology and function changes, causing serious consequences such as renal tubulointerstitial inflammation, kidney stones, renal fibrosis and polycystic kidney disease (64). The study of 266 patients with hyperuricemia found that the incidence of nephropathy was 15.11%, while the incidence of nephropathy was only 2.19% in the population with normal serum uric acid level (65). Another study among 190 patients with chronic gout found that the incidence rate of renal damage was 86.13%, significantly higher than 7.14% in the control group, suggesting that hyperuricemia was closely related to the incidence of renal damage and was another risk factor for kidney diseases (66).

At present, it is believed that hyperuricemia induced kidney injury is mainly related to hyperuricemia induced RAAS hyperfunction, inflammatory reaction, renal microvascular injury and so on, but the exact mechanism remains unclear. Affiliated Bao’an Hospital of Shenzhen conducted a cohort study among control, hyperuricemia and gouty nephropathy patients. The results showed that the expression of the NLRP3 inflammasome in peripheral blood mononuclear cells, and the levels of IL-1β and IL-18 in the plasma were upregulated in the gouty nephropathy group compared with the control and hyperuricemia groups (67). In rats with hyperuricemia and dyslipidemia induced by fructose, NLRP3 inflammasome in kidney tissues was activated, which manifested by overexpression of NLRP3, ASC and caspase-1, resulting in excessive production of IL-1β, IL-18, IL-6 (68). Using the CRISPR/Cas9 system to functionally disrupt expression of urate oxidase (UOX) in Wistar rats spontaneously and persistently increased serum uric acid level compared with wild-type rats. UOX-KO rats established increased interstitial fibrosis, macrophage infiltration, increased expression of NLRP3 and IL-1β, and activation signaling pathways associated with autophagy, indicating that autophagy and NLRP3-dependent inflammation played crucial role in the development of hyperuricemia induced kidney injury (69). The activation of NLRP3 inflammasome has been proved to be a target of hyperuricemia induced renal injury. A large number of natural extracts have been found to improve renal function by inhibiting the activity of NLRP3 inflammasome in hyperuricemia (70–73).

Although there are a lot of epidemiological and experimental research reports on the relationship between uric acid and kidney injury, the underlying pathological mechanism still needs further research. The mechanism of hyperuricemia-induced renal injury has a wide range of cross-talks. The activation of NLRP3 inflammasome plays a complex and important role in promoting the occurrence and development of renal disease. The mechanism of its interaction with other factors still needs to be further explored.

### Hyperhomocysteinemia

Hyperhomocysteinemia (hHcys) is a disease characterized by elevated homocysteine in the blood, which has been recognized as one of the important risk factors of kidney disease. HHcys can cause anabolism of cholesterol and triglycerides, impaired endothelial function, thrombosis, and monocyte activation (74). Hyperhomocysteinemia is present in 85% of patients with chronic renal failure, and persists after the initiation of dialysis or kidney transplantation (75).

In 2012, Zhang et al. firstly discovered that all the components of NLRP3 inflammasome were existed in podocytes and were significantly evaluated by the treatment with L-homocysteine (L-Hcys) (76). Silencing the ASC gene or inhibiting caspase-1 activity could alleviate podocyte injury and improve glomerulosclerosis in mice with hHcys (76). Podocin, nephrin and desmin are critical markers of podocyte injury. Another study demonstrated that in folate free (FF) diet induced hHcys mice, NLRP3−/− mice showed increased protein level of podocin and nephrin but decreased expression of desmin compared to wild-type mice, indicating the potential pathogenic effects of NLRP3 inflammasome activation (77). The production of ROS plays an important role in the activation of NLRP3 inflammasome in hHcys-induced kidney injury. Nicotinamide adenine dinucleotide phosphate (NADPH) oxidase is considered to be the main source of superoxide in the kidney. NADPH oxidase inhibition (NOX) reversed the upregulated protein levels of NLRP3, ASC and caspase-1 stimulated by Hcys in mouse podocytes (78). *In vivo*, NOX inhibition also protected glomeruli and podocytes from hHcys-induced damage, which was manifested by reduced proteinuria and glomerular sclerosis (78). TEMPOL is a recognized antioxidant. In hHcys mice, the treatment of TEMPOL reduced colocalization of NLRP3 with ASC, caspase-1 activation and as well as IL-1β production, suggesting the treatment inhibited the activation of NLRP3 inflammasome (79). Meanwhile, the glomerular injury induced by hHcys has also been improved (79). As in other metabolic-associated kidney diseases mentioned above, the binding of TXNIP to NLRP3 is a key signaling mechanism in hHcys-induced kidney injury as well. Inhibition of TXNIP by verapamil or TXNIP shRNA transfection broke the binding and disrupted the formation of glomerular inflammasome (80).

As a crucial role in the pathogenic process of hHcys-induced kidney injury, NLRP3 inflammasome has been regarded as a novel target for the treatment of glomerular injury in hHcys. Many compounds with anti-inflammatory properties, such as anandamide, DHA metabolites-resolvins, resolvin D1 (RvD1) and 17S-hydroxy DHA (17SHDHA), blocked podocyte injury and glomerular sclerosis during hHcys *via* the suppression of NLRP3 inflammasome activity (81–83).

## Conclusion

MS is a group of clinical syndromes of chronic inflammation and metabolic disorders caused by insulin resistance. With the improvement of the economic level and the spread of unhealthy lifestyles, the prevalence of MS is on the rise globally, especially in developing countries and regions. Recent studies have found that MS is an independent risk factor for CKD. The pathogenesis of kidney damage caused by MS is related to poor primary disease control, insulin resistance, chronic inflammation, and endothelial function damage. NLRP3 inflammasome is the sensor of the innate immune system that initiates inflammatory response to stimulations, and participates in the occurrence and development of various metabolic diseases and kidney injury (**Table 1**). As we mentioned above, a variety of metabolic disorders leads to the activation of NLRP3 inflammasome in the kidney. The activation of NLRP3 inflammasome aggravates renal inflammatory infiltration and tissue damage through many pathways including autophagy, inflammatory factor release and EMT. At the same time, factors related to tissue damage, such as ROS, autophagy related molecules continue to stimulate the activation of NLRP3 inflammasome and impair renal function. Therefore, the activation of inflammasome and kidney injury are mutually reinforcing.

**Table 1:** The role of NLRP3 inflammasome in the kidney under different metabolic disorders.

Metabolic factor	NLRP3 state	Function
Hyperglycemia	activate	podocyte lose, glomerulosclerosis and tubulointerstitial fibrosis
Hypertension	activate	hypertension, proteinuria, interstitial inflammation and fibrosis
Obesity	activate	dyslipidemia, podocyte injury, cell death and apoptosis
Hyperuricemia	activate	interstitial fibrosis and macrophage infiltration
Hyperhomocysteinemia	activate	proteinuria and glomerular sclerosis

Because inflammasomes are intracellular recognition receptors, scientists believe that there may be common factors for their activation. In the kidney damage caused by different metabolic factors, there are currently three recognized activation pathways, namely the outflow of potassium ions, the release of ROS, and the rupture of lysosomes. But for each metabolic factor, specific activators are also found. For example, in DN, NLRP3 inflammasome can also be activated by endoplasmic reticulum stress and autophagy.

Accordingly, to explore the specific mechanism and important role of NLRP3 in kidney injury induced by metabolic disorders will provide new ideas and directions for the prevention and treatment of metabolic-associated kidney diseases.

## Author Contributions

CZ and X-FM conceived and designed the manuscript. WX did literature searching, drafted the manuscript, and drew the figures. CZ and X-FM reviewed and revised the article. All authors contributed to the article and approved the submitted version.

## Funding

This work was supported by Grants from the National Natural Science Foundation of China (81961138007, 81974096, 81770711), and program for HUST Academic Frontier Youth Team (2017QYTD20).

## Conflict of Interest

The authors declare that the research was conducted in the absence of any commercial or financial relationships that could be construed as a potential conflict of interest.
